# Bringing *PLOS Genetics* Editors to Preprint Servers

**DOI:** 10.1371/journal.pgen.1006448

**Published:** 2016-12-01

**Authors:** Gregory S. Barsh, Casey M. Bergman, Christopher D. Brown, Nadia D. Singh, Gregory P. Copenhaver

**Affiliations:** 1 HudsonAlpha Institute for Biotechnology, Huntsville, Alabama, United States of America; 2 Department of Genetics, Stanford University School of Medicine, Stanford, California, United States of America; 3 Department of Genetics, University of Georgia, Athens, Georgia, United States of America; 4 Department of Genetics, University of Pennsylvania, Philadelphia, Pennsylvania, United States of America; 5 Program in Genetics, Department of Biological Sciences, North Carolina State University, Raleigh, North Carolina, United States of America; 6 Department of Biology and the Integrative Program for Biological and Genome Sciences, University of North Carolina at Chapel Hill, Chapel Hill, United States of America

What’s the first thing you do after making a cool new discovery? If you’re like us, you run up and down the hallway, propelled by excitement, eager to show your latest result to your colleagues. But the hallway is a pretty limited audience, so we soon turn to publishing our work in a peer-reviewed journal to show it to the whole world (assuming it is an open-access journal). That’s when the fun of discovery can come to a screeching halt and turn into dreary hours of formatting and online form submission only to wait weeks, if not months, for your manuscript to wend its way through the peer-review system. Preprint servers (PPS) can short-circuit those cheerless steps, at least for a time, and allow the fruits of your labor to be seen immediately by all who are interested. In addition to increasing the visibility of authors' work, PPS provide opportunities for journals to identify manuscripts that are good fit for their audience. In that vein, *PLOS Genetics* is pleased to announce a new initiative to use PPS for identifying and soliciting manuscripts, as part of PLOS’ overall mission to improve the efficiency and accessibility of science communication (and, of course, to make the process less cheerless for authors). As part of that effort, we now have a dedicated team of editors who will focus on identifying manuscripts on PPS that are potentially suitable for publication in *PLOS Genetics*.

PPS are online databases that store and distribute scholarly works prior to publication. Once you’ve written your manuscript as you normally would, you can upload it to a PPS and submit it whenever and wherever you like for peer-review and publication (most publisher’s policies are compatible with preprints; see Wikipedia’s list of academic journals by preprint policy to check before submitting). In the interim, the research community can benefit from your findings, leave helpful comments, and make suggestions about how your study can be improved. There is growing recognition at *PLOS Genetics* (and in the scientific community more broadly) that PPS like arχiv (https://arxiv.org/) and bioRχiv (http://biorxiv.org/) play an increasingly important role in the scientific publishing ecology. Concomitant with the rise in popularity of PPS, a marketplace of preprints is now emerging. Publishers can now seek out and identify high-quality manuscripts deposited in PPS and compete to invite the authors to submit their work to a particular journal. We at *PLOS Genetics* believe this competitive intellectual marketplace will benefit working scientists in many ways. Most importantly, having journals recruit manuscripts will reduce the time spent getting your paper into review at a suitable journal, ultimately shortening the time it takes for your work to reach your target readership and thereby extending its window of influence. We also believe that preprints will continue to increase in value as professional currency for hiring committees, grant panels, and tenure and promotion committees. This trend can already be seen with the recent National Institutes of Health biosketch format rules (NOT-OD-16-086), which now allow preprints to be listed. We hope that our new initiative to solicit manuscripts from PPS will offer a clear incentive for more authors to embrace the use of preprints in biology.

Our Preprint Editors will use a combination of their own judgment, a reading of the comments posted to the preprint server, and some automated tools to identify candidate manuscripts. They will then initiate a rapid consultation with editors on our board who have relevant specific expertise to determine if the manuscript has the qualities that would make it successful at our journal—it reports a significant advance in its field, would be of interest to the broad genetics community, and is technically excellent. After identifying exciting candidate manuscripts, the Preprint Editors will contact the authors and invite them to submit their work to *PLOS Genetics*. For authors using the bioRχiv PPS, a direct transfer mechanism (dubbed B2J) makes initial submission to *PLOS Genetics* (and over 35 other journals) as easy as a single click. As this is a new initiative, we expect the process for soliciting manuscripts from PPS to evolve over time, and we welcome feedback about how to make this system work best for the *PLOS Genetics* community.

We acknowledge that PPS will not be right for every manuscript or every author, so we want to reassure readers and authors that nothing at the journal is going away. We’ll still be doing business as usual, but now we’ll now be doing it with an extra option that has an eye towards the future. We at *PLOS Genetics* are excited to grow with the needs of the scientific community, and we hope you’ll be as excited as authors to join us. We look forward to seeing your manuscripts on PPS so *PLOS Genetics* can have the chance to compete for your best work.

